# Personality traits among Moroccan officials in the Rabat-Sale-Kenitra Region

**DOI:** 10.1192/j.eurpsy.2023.2060

**Published:** 2023-07-19

**Authors:** E. Drissi, F. Z. azzaoui, H. Hami, A. Ahami, S. Boulbaroud

**Affiliations:** 1 Biology, ibn tofail university, kenitra; 2 Biology, Polydisciplinary Faculty, Sultan Moulay Sliman University, benimellaj, Morocco

## Abstract

**Introduction:**

Public servants, because of the nature of their work, are under tremendous pressure. Hence the need to study the dominant personality traits within this sample.

**Objectives:**

Demonstrate the domination of certain personality dimensions over others in this population.

The aim of this research is to study the five major personality traits in a sample of officials in the Rabat-Sale-Kenitra Region, Morocco.

**Methods:**

This is a cross-sectional epidemiological study that involved 387 individuals, including 55.8% (n=216) Male and 44.2% (n=171) Female with an average age of 32.75± 9.79. The personality traits were assessed using the Big Five test.

**Results:**

The results show that, 76.74% of our study subjects have a high score of Agreeableness, while 23.26% have a low score. 59.69% of our sample has a high score in Extravesion, while 40.31% have a low one. Slight increase in the percentage that has a high score in Neurosis compared to those that have a low score with 51.94% and 48.06%. 78.29% of our sample have a high Consciousness score, while 21.71% have a low one. Concerning Openness, 79.84% of the participants have a high score and only 20.16 have a low score.

**Conclusions:**

This study sample is characterized by the dominance of three main traits, Agreeableness, Consciousness and Openness. Moreover, this study has shed light on the fact that the Neurosis trait is dominant in almost half of our sample. However, considering this study concerned only one region, it would be interesting to widen the geography of the survey to acquire more exhaustive results.

**Disclosure of Interest:**

None Declared

